# Inflammation-Mediated Angiogenesis in Ischemic Stroke

**DOI:** 10.3389/fncel.2021.652647

**Published:** 2021-04-21

**Authors:** Hua Zhu, Yonggang Zhang, Yi Zhong, Yingze Ye, Xinyao Hu, Lijuan Gu, Xiaoxing Xiong

**Affiliations:** ^1^Department of Neurosurgery, Renmin Hospital of Wuhan University, Wuhan, China; ^2^Central Laboratory, Renmin Hospital of Wuhan University, Wuhan, China; ^3^Department of Oncology, Renmin Hospital of Wuhan University, Wuhan, China; ^4^Department of Anesthesiology, Renmin Hospital of Wuhan University, Wuhan, China

**Keywords:** angiogenesis, stroke, immune cells, inflammation, inflammatory cytokine

## Abstract

Stroke is the leading cause of disability and mortality in the world, but the pathogenesis of ischemic stroke (IS) is not completely clear and treatments are limited. Mounting evidence indicate that neovascularization is a critical defensive reaction to hypoxia that modulates the process of long-term neurologic recovery after IS. Angiogenesis is a complex process in which the original endothelial cells in blood vessels are differentiated, proliferated, migrated, and finally remolded into new blood vessels. Many immune cells and cytokines, as well as growth factors, are directly or indirectly involved in the regulation of angiogenesis. Inflammatory cells can affect endothelial cell proliferation, migration, and activation by secreting a variety of cytokines via various inflammation-relative signaling pathways and thus participate in the process of angiogenesis. However, the mechanism of inflammation-mediated angiogenesis has not been fully elucidated. Hence, this review aimed to discuss the mechanism of inflammation-mediated angiogenesis in IS and to provide new ideas for clinical treatment of IS.

## Introduction

Ischemic stroke (IS) is one of the diseases with the highest incidence and disability rate in the world. Currently, the effective treatment measure for IS is thrombolytic therapy with tissue plasminogen activator to recirculate cerebral blood flow. However, due to the limitation of the treatment time window, only a small number of patients are suited to this treatment (Zhou et al., 2019). Thus, clarifying the pathogenesis and exploring new therapies are urgently needed to improve outcomes. Normal collateral circulation can effectively improve the blood flow supply of brain tissue. The extension of neovascularization from normal brain tissue to the ischemic penumbra and central necrotic area can improve the blood perfusion of the surrounding ischemic area, reduce the volume of cerebral infarction, remold the nerve structure, and restore the central nervous system (CNS) function. Therefore, more and more studies have been conducted to promote the formation of neovascularization in the ischemic and surrounding areas after IS. Immune cells, such as mononuclear macrophages, T cells, and microglia, are involved in angiogenesis after stroke through different mechanisms. Here, we focus on the role of immune cells and related cytokines in neurovascular remodeling after IS and discuss the potential therapies for stroke that target neurovascular remodeling.

## Overview of Angiogenesis

Angiogenesis, known as the formation of neovascularization that is induced by the sprouting of endothelial cells (ECs) from preexisting vessels, occurs under a variety of physiological (e.g., reproduction) and pathological (e.g., IS) conditions (Potente et al., 2011). During the sprouting process, ECs degrade the basement membrane, migrate into adjacent tissues, proliferate, and aggregate into tubes to form new blood vessels and establish blood flow. Basement membrane starts to form under the joint action of pericytes, ECs, and the microenvironment and further promotes the maturation and stability of new tubes, then finally completes the vascular remodeling (Marti et al., 2000). The proliferation and migration of ECs, increased vascular permeability, and the degradation of the surrounding stroma, as well as the stabilization and cessation of angiogenesis, are essential steps of angiogenesis (Hayashi et al., 2003). Under physiological state, angiogenesis is in the dynamic balance of the angiogenesis-promoting factor and the inhibiting factor. Once the balance is broken, insufficient angiogenesis will lead to slow wound healing, such as stroke. Angiogenesis can salvage cerebral blood flow and supply nutrients/oxygen to the ischemic brain tissue, finally rescuing grievous neurons and impaired tissues (Krupinski et al., 1994). An elevated vascular density in the brain ischemic penumbra region was observed 3 days after IS in animal and human brain specimens (Hayashi et al., 2003). Excessive angiogenesis is commonly seen in the occurrence, development, and metastasis of tumors.

## The Role of Angiogenesis in IS

Stroke is usually the result of focal cerebral ischemia caused by cerebral stenosis or occlusion. The blood flow of the brain tissue in the cerebrovascular region drops sharply, leading to a sharp decrease in the supply of oxygen and nutrients and eventually leading to tissue cell damage and even death. The potential mechanism of compensatory increased oxygen supply to ischemic brain tissue is the induction of new vessels. Neovascularization in the ischemic infarction area regulates the cerebral blood flow, neuron repair and regeneration, and the degree of functional recovery of patients as well as the functional reconstruction or rebuilding of synaptic connections between nerve cells (Pan et al., 2010). The collateral circulation in the initial phase of IS relies on the opening of a preexisting vascular network, while in the later period, it mainly depends on the formation of new blood vessels. New blood vessels begin to be observed at 3 days after IS and increase continuously for 21 days or more (del Zoppo and Mabuchi, 2003). Angiogenesis after stroke has therapeutic potential. Postmortem examination of the cerebral tissue of stroke patients with different survival times showed that the density of microvessels on the infarcted side was notably higher than that of the normal hemisphere on the opposite side. The number of new blood vessels around the infarct is positively correlated with the survival rate, survival time, and neurological function recovery of stroke patients (Krupinski et al., 1994). Other studies have found that patients with dementia show severe decreased cerebral blood flow in non-infarcted areas, indicating that angiogenesis can improve patients' daily activities by increasing cerebral blood flow (Schmidt et al., 2000). In a mouse model of middle cerebral artery occlusion (MCAO), proliferating ECs were found in the ischemic penumbra and were associated with increased vascular density (Kim et al., 2020). The ischemic penumbra, a structurally intact but functionally impaired region around the ischemic core area after cerebral infarction, is the main target of therapeutic intervention. Further exploration of the mechanism of angiogenesis after IS is conducive to research on more treatment options.

## Inflammatory-Mediated Angiogenesis After IS

Previous studies have identified that various immune cell subgroups regulate angiogenesis, such as natural killer (NK) cells, T helper 17 (Th17) cells, regulatory T lymphocytes (Tregs), and a functional subset of macrophages (M2 macrophages) (la Sala et al., 2012). Numerous angiogenic growth factors, commonly associated with inflammatory response, including vascular endothelial growth factor (VEGF), tumor necrosis factor-α (TNF-α), monocyte chemotactic protein-1 (MCP-1/CCL2), transforming growth factor-β (TGF-β), fibroblast growth factor (FGF), granulocyte-macrophage colony-stimulating factor (GM-CSF)/granulocyte colony-stimulating factor (G-CSF), hepatocyte growth factor (HGF), platelet-derived growth factor (PDGF), and interleukins (ILs), play crucial roles in inflammatory-mediated angiogenesis after IS (Schierling et al., 2009). The molecular mechanisms of inflammation-mediated angiogenesis involved in IS are discussed below.

## The Role of Immune Cell Subsets and Inflammatory Cells in Angiogenesis After IS

### NK Cells

NK cells, an important subgroup of immune cells, infiltrate into local ischemic brain tissue and act as a bridge between the immune system and the CNS in IS patients. NK cells are closely associated with immunosuppression, inflammation, and infection after IS (Chen et al., 2019). Studies have confirmed that in pathological and physiological conditions, NK cells produce cytokines that promote angiogenesis and independently regulate angiogenesis (Ribatti et al., 2019). IS leads to other complications, such as cerebral small vessel disease. In the cerebral microvasculature of spontaneously hypertensive (a model for early-onset cerebral small vessel disease) rats after IS, NK cells infiltrated markedly in the cerebral microvessels, leading to inflammatory reactions and hypertension-associated cognitive dysfunction (Kaiser et al., 2014). The activated NK cells are involved in endothelial and vascular function deficits by promoting the expression of interferon gamma (IFN-γ) (Kossmann et al., 2013). Angiogenesis is an important compensation method for arterial obstruction. In addition, NK cells play crucial roles in cerebrovascular formation in hindlimb ischemia mice (van Weel et al., 2007).

### CD4^+^ T Cells

CD4^+^ T cells (consisting of Th1, Th2, Th17, and Tregs), which are responsible for inflammatory response, boost arteriogenesis in a murine model of hindlimb ischemia (Nossent et al., 2017). Moreover, CD4^+^ T cells play essential roles in activating and polarizing macrophages, which have been demonstrated to have the ability to promote angiogenesis (Zhang et al., 2020). Th17 promotes angiogenesis and increases cerebral blood flow in the ischemic penumbra, partly owing to its potent effect in promoting the migration and sprouting of ECs (Kwee et al., 2018). The study has shown that hyperforin could promote subependymal ventricular zone angiogenesis, neurogenesis, and functional recovery through suppressing Th1 cell differentiation while facilitating Th2/Treg cell differentiation in the ischemic area, which is mediated by astrocyte IL-6 (Yao et al., 2019). Hence, it indicates that the infiltration of Th2 cells into the ischemic hemisphere increased Th2-derived cytokine IL-4, increased Treg-derived IL-10 and TGF-β, as well as decreased Th1 and Th1-derived cytokine IFN-γ, which are all key events that promote angiogenesis during stroke recovery.

### Microglia/Macrophage Cells

Resident microglia and infiltrated macrophages activate the immune defense in the CNS to regulate the inflammatory condition dynamically in the injured brain (Zhu et al., 2019). Microglia/macrophages can polarize into the M1 and M2 phenotypes to participate in IS damage or repair (Liu et al., 2018). M1 is known to be classically activated and to exert pro-inflammatory effects, whereas M2 is alternatively activated and plays an anti-inflammatory role in IS. M2 macrophages, a more heterogeneous phenotype, can be further divided into M2a and M2c according to their function. The M2a subset activated by IL-4 is regarded as the wound healing macrophages that also belong to the alternatively activated macrophages; the M2c subgroup activated by numerous stimulants, including glucocorticoids, immune complexes, and IL-10, as well as prostaglandins, is known as the regulatory macrophages.

Microglial/macrophage cells are also crucial to angiogenesis after IS. Evidence shows that increased microvessel density after IS was detected only in the regions also containing macrophages, suggesting that angiogenesis is related to the increased number of macrophages (Manoonkitiwongsa et al., 2001). Moreover, the brain may synthesize microvessels and open capillaries needed for macrophage infiltration for the destruction and removal of necrotic brain tissue via the angiogenic signaling cascade. Microglial activation and the related neuroinflammation in the ischemic penumbra region were alleviated by polarizing the microglia toward the M2 phenotype (Shang et al., 2020). Previous studies have indicated that M2 microglia/macrophages have a higher potential to promote angiogenesis than other subgroups. Moreover, FGF signaling for M2a- and placenta growth factor (PlGF) signaling for M2c-induced angiogenesis may be the possible working mechanisms (Jetten et al., 2014). Hence, further researches are needed to illustrate the exact mechanism of angiogenesis induced by M2.

### Brain Pericytes

Brain pericytes are perivascular cells that play a critical role in the regulation of capillary function and the development of cerebral microcirculation, and their location puts them in a crucial position to regulate the neuroinflammation at the neurovascular unit (NVU), which consists of neuronal cells, pericytes, ECs, astrocytes, microglia, and the extracellular matrix (Bell et al., 2010; Muoio et al., 2014). Brain pericytes are involved in a variety of brain functions, such as the generation and stabilization of the blood–brain barrier (BBB), brain inflammation, and tissue regeneration, as well as the propagation of CNS and peripheral inflammation (Umehara et al., 2018). Moreover, pericytes recently have been verified to exert many functions of immunomodulatory cells, including producing and responding to inflammatory cytokines, presenting antigen, and exhibiting phagocytosis. Hence, brain pericytes are increasingly considered to be mediators of neuroinflammation (Rustenhoven et al., 2017), thereby participating in inflammatory reaction during IS.

Previous studies have shown that the continuous constriction and necrocytosis of pericytes in the hyperacute phase leads to the no-reflow phenomenon, and the inflammatory responses and the separation of pericytes in the acute phase may exacerbate the BBB injury (Uemura et al., 2020). However, pericytes also exert a neuroprotective role via releasing neurotrophins, EC protection, and BBB stabilization (Franco et al., 2011; Winkler et al., 2011; Ishitsuka et al., 2012). Pericytes play a powerful role in neural restoration after IS via promoting angiogenesis and neurogenesis. The bidirectional ECs–pericytes signaling is essential during angiogenesis (Stapor et al., 2014). Firstly, after being stimulated by pro-angiogenic factors, pericytes produce numerous matrix metalloproteinases (MMPs) (MMP-2, MMP-3, and MMP-9) to degrade the components of the basement membrane, resulting in the separation of pericytes and the succeeding release of ECs (Virgintino et al., 2007; Candelario-Jalil et al., 2009; Potente et al., 2011). Furthermore, increased vascular permeability promotes plasma protein extravasation and provides a temporary scaffold for vascular germination (Carmeliet and Collen, 2000). In addition, pericytes generate VEGF-A, which activates ECs to protrude filopodia, leading to the formation of tip cells (Jakobsson et al., 2010; Franco et al., 2011). Then, stalk cells multiply to support the extension of sprouts and construct a vascular vessel (Eilken and Adams, 2010). Meanwhile, pericytes adhere to ECs, regulate the sediment of the extracellular matrix and the modeling of the inter-endothelial tight junctions, and stabilize neovascularization. Lastly, tip cells anastomose with adjacent sprouts to form microvascular rings (Jain, 2003). Then, pericytes are transformed to suppress EC multiplication and reestablish the quiescence state of ECs to stop angiogenesis (Yang et al., 2017). The primary pericyte-associated pathways propelling neovascularization after IS include the PDGF-BB/PDGFRβ pathway (Gaengel et al., 2009), VEGF-A/VEGFR2 pathway (Greenberg and Jin, 2005), TGF-β/TGFRβ2 pathway (Walshe et al., 2009; Li et al., 2011), Notch pathway (Kume, 2012), and the Ang/Tie2 pathway (Armulik et al., 2011). Here, we have roughly concluded the mechanism of pericytes involved in angiogenesis after IS; further studies are needed to elucidate the mechanism of angiogenesis after IS and to explore feasible pro-angiogenic therapies.

## The Role of Inflammatory Cytokines in Angiogenesis After IS

### VEGF

VEGF, diffusely distributed in brain tissue, has essential effects in brain inflammation through promoting inflammatory cell recruitment and adjusting angiopoietin II (Ang II) secretion (Yin et al., 2020). VEGFs and their receptors (VEGFRs) are also pivotal regulators of blood vessel formation. VEGF is mainly divided into matrix-bound and soluble subtypes. Matrix-bound subtypes stimulate more vessel branching, while soluble VEGF facilitates vascular dilation (Carmeliet, 2003). The VEGF family consists of seven subsets of ligands: VEGF-A, VEGF-B, VEGF-C, VEGF-D, VEGF-E, VEGF-F, and PlGF. PlGF, VEGF-A, and VEGF-B are required for angiogenesis (Takahashi and Shibuya, 2005). VEGF-A (also known as VEGF) is the major member of the VEGF family and promotes angiogenesis via binding to VEGF receptor-2 (VEGFR-2) (Zhong et al., 2020). VEGF-B, functionally and structurally related to VEGF-A and PlGF, regulates the bioavailability of VEGF-A (Olofsson et al., 1996). The binding of VEGFs to their receptors on the cytomembrane of ECs activates the mitogen-activated protein kinase (MAPK)/extracellular signal-regulated kinase (ERK) 1/2 and phosphatidylinositol-3 kinase (PI3K)/protein kinase B (AKT) pathways, leading to EC survival, proliferation, differentiation, migration, and tube formation (Assareh et al., 2019). Deficiency of VEGF and/or VEGFR-2 leads to vascular formation defects. In addition, the biological effects of VEGFR-2 may vary greatly due to its subcellular localization. For example, VEGFR-2 must signal from the endocellular compartments when VEGF induces arterial morphogenesis (Lanahan et al., 2010). In addition, autocrine VEGF, secreted by ECs, maintains vascular stabilization (Lee et al., 2007), whereas paracrine VEGF, produced by myeloid, tumor, or stromal cells, renders tumor vessels abnormal and increase vessel branching (Stockmann et al., 2008). Endostatin inhibited VEGF-induced endothelial tube formation by stabilizing the newly formed endothelial tubes (Ergün et al., 2001). Hence, endostatin may be a new anti-angiogenic agent in tumor therapy.

As a potent angiogenic factor, VEGF also has neuroprotective activity. A previous study has illustrated that VEGF promotes neurovascular remodeling in the ischemic hemisphere, contralesional corticobulbar plasticity, and neurological recovery. This brain remodeling by VEGF may be achieved by the deactivation of MMP9, activation of c-Jun, and the suppression of caspase-3-dependent apoptotic cell death as well as the downregulation of growth inhibitory proteoglycans and guidance molecules (Reitmeir et al., 2012). In addition, VEGF has been reported to attenuate the CD45^+^ leukocyte infiltrates at 14 days after ischemia and to diminish the activation of the microglia. Intracerebroventricularly delivered VEGF promotes contralesional corticorubral plasticity through its anti-inflammatory action via the downregulation of a broad set of inflammatory cytokines and chemokines in both brain hemispheres (Herz et al., 2012). Although the overexpression of VEGF exerts neuroprotection after IS, VEGF facilitates a hemodynamic steal phenomena with reduced blood flow in ischemic areas and increased flow values only outside the MCA territory. The overexpression of VEGF can significantly alleviate neurological deficits and infarct volume and reduce the disseminated neuronal injury and caspase-3 activity, confirming that VEGF has neuroprotective properties. However, a mild blood–brain barrier leakage has been reported to be induced by VEGF (Wang et al., 2005). Hence, the overexpression of VEGF may worsen rather than improve cerebral hemodynamics after IS. The survival-enhancing effect of VEGF is purchased at the expense of increased BBB permeability, which may be regulated by the PI3K/AKT pathway (Kilic et al., 2006). Taken together, the neuroprotective functions of VEGF cannot be easily exploited without accepting the adverse consequences of increased vascular permeability.

### MCP-1

MCP-1, also known as CCL2, a key chemokine that regulates the activation and recruitment of monocytes and microglias as well as T cells, has been implicated in inflammatory and angioproliferative CNS disease (Zhang and Luo, 2019). Both MCP-1 and its receptor C–C chemokine receptor type 2 (CCR2) expressed in the brain are crucial regulators of CNS inflammation and activation of the microglia, involved in various neuroinflammatory diseases (Hinojosa et al., 2011). During inflammatory response, MCP-1 attracts peripheral monocytes to the brain and regulates Th cell development by stimulating Th2 polarization (Saoud et al., 2019). Coincidentally, we have discussed above that the infiltration of Th2 cells to the ischemic hemisphere and the increased Th2-derived cytokine IL-4 can promote angiogenesis during stroke recovery. MCP-1 is also acknowledged as an angiogenic chemokine. However, the mechanism of angiogenesis mediated by MCP-1 has not been fully elucidated. Previous studies indicate that MCP-1-mediated angiogenesis mainly consists of two successive steps: the expression of VEGF-A gene induced by MCP-1 and the subsequent neovascularization induced by VEGF-A. MCP-1 upregulates the expression of the hypoxia-inducible factor 1 alpha (HIF-1α) gene in human aortic ECs, which induces VEGF-A expression in the human aortic wall and ECs via activating MAPK (Hong et al., 2005). A previous study has illustrated that VEGF also induces the expression of MCP-1, mainly through activating NF-kB and the activator protein 1 (AP-1) in retinal ECs (Marumo et al., 1999). MCP-1-mediated angiogenesis is also induced by MCP-1-induced protein (MCPIP), at least partly via the transcriptional activation of cadherin (cdh) 12 and cdh19. Additionally, MCP-1 is involved in neurological recovery after IS and delivers varieties of cells into the brain through MCP-1/CCR2 interplay. Moreover, after intravenous transplantation of human umbilical cord-derived mesenchymal stem cells (hUC-MSCs) with overexpression of MCP-1 in MCAO rats, angiogenesis was promoted in the ischemic penumbra region (Lee et al., 2020). This indicates that MCP-1-overexpressing hUC-MSCs distinctly repaired functional deficits in rats with MCAO by accelerating persistent increases in MCP-1 levels in brain tissue, promoting neurogenesis and angiogenesis and inhibiting neuroinflammation. These results demonstrate that MCP-1-overexpressing MSCs might be an alternative therapeutic method for cell therapy of IS.

### TNF-α

TNF-α, a cytokine with diverse pro-inflammatory actions, plays crucial roles in both pathological and physiological conditions (Saha and Smith, 2018). TNF-α is induced in the ischemic cerebral tissue within 1 h, peaks at 6–10 h, and subsides 1–2 days post-IS (Liu et al., 1994). Increased TNF-α exerts both neuroprotective and neurotoxic effects after IS (Wilde et al., 2000). TNF-α acts via interacting with two kinds of receptors, TNF receptor 1 (TNFR1) and TNFR2, which can mediate hindlimb ischemia-induced angiogenesis (Luo et al., 2006). Researches in neuroprotection have shown that TNFR1 targeting the erythropoietin receptor (EPOR) and VEGF can alleviate cortical neuronal injury after IS (Taoufik et al., 2008). Moreover, the interplay between TNF-α and TNFR1 primes brain ECs for erythropoietin (EPO)-enhanced angiogenesis by the upregulation of EPOR that amplifies the effect of EPO on the activation of VEGF/VEGFR2 and the angiopoietin 1 (Ang1)/angiopoietin receptor (Tie2) signaling pathway (Wang et al., 2011). In addition, TNF/TNFR1 can act directly on ECs to regulate angiogenesis via activating VEGF signaling (Sugano et al., 2004). TNF also activates various pathways, such as NF-κB and AKT. The NF-κB activated by TNF may trigger the activation of AKT signaling, whereas both NF-κB and PI3K/AKT pathways are essential for the TNF/TNFR1-upregulated expression of EPOR (Wang et al., 2011). These evidences certify the network between TNF/TNFR1 and EPOR to coordinate the occurrence of neovascularization in brain ECs. Additionally, other studies illuminated that IS-induced angiogenesis relies on the TNFR1-mediated upregulation of α5β1 and αVβ3 integrins (Huang et al., 2016).

### TGF-β

The TGF-βs consist of three mammalian subtypes (TGF-β1, TGF-β2, and TGF-β3). The component of TGF-β signaling transduction, including the serine kinase-type receptor on the cytomembrane, exists in the CNS. TGF-βs are pluripotent cytokines whose potential neuroprotective actions are gradually acknowledged. Actually, the expression of TGF-β can be activated following various brain injuries. The neuroprotective action of TGF-β is mostly induced by IS (Dobolyi et al., 2012). Injury in animal models of local and global cerebral ischemia was observed to be suppressed by TGF-βs. A previous study has shown that TGF-βs inhibit microglias, thereby exerting an anti-inflammatory effect (Lund et al., 2018). Since the microglia is the main source of TGF-β1 in the CNS, they may have an autoinhibitory effect on the microglia (Lenzlinger et al., 2001). The potential neuroprotective mechanisms of TGF-βs include anti-inflammatory, anti-excitotoxic, and anti-apoptotic abilities and the facilitation of angiogenesis, neurogenesis, and scar formation.

The secretion of TGF-β1 was proven following IS damage. The expression of TGF-β1 in the ischemic penumbra region was significantly elevated after MCAO in baboons and rats (Ali et al., 2001; Vincze et al., 2010). Moreover, increased levels of TGF-β1 were also found in human brain following IS (Krupinski et al., 1996). Activated microglia/macrophages have been demonstrated to be the primary sources of TGF-β1 messenger RNA (mRNA) after focal ischemic attack (Lehrmann et al., 1998; Doyle et al., 2010). In addition, astrocytes and neuronal cells may also contribute to the upregulated TGF-β1 expression after IS (Zhu et al., 2001; Malik et al., 2020).

TGF-β is known to facilitate angiogenesis. After subcutaneous injection of TGF-β in neonatal mice, collagen production by fibroblasts and increased angiogenesis were observed at the injection site (Roberts et al., 1986). A previous study has demonstrated that TGF-β1 can enhance the actions of other growth factors. In fact, TGF-β1 amplified the therapeutic action of VEGF-induced angiogenesis in patients with peritoneal dialysis (Kariya et al., 2018). Using a two-dimensional Petri dish and three-dimensional hydrated collagen gel cultures, a low concentration of TGF-β1 amplified the stimulating effect of basic fibroblast growth factor. TGF-β1 also promoted the proliferation of ECs in the disc angiogenesis system (Gajdusek et al., 1993). To explore the effect of endogenic TGF-β on spontaneous vascular growth of wound healing, the application of an anti-TGF-β antibody suppressed the spontaneous angiogenesis lower than the control group (Fajardo et al., 1996). Therefore, endogenous TGF-β exerts the effect of promoting spontaneous angiogenesis. Additionally, deficiency of the different components of TGF-β signaling has verified that TGF-β is essential for angiogenesis, and TGF-β receptor mutations are associated with a vascular disease called hereditary hemorrhagic telangiectasia (Bertolino et al., 2005). What is more, in ischemic rat brain following MCAO, leucine-rich α2-glycoprotein 1 (LRG1) may protect the rat brain tissue from ischemic attack through accelerating angiogenesis via upregulating the TGF-β1 pathway (Meng et al., 2016). It has been suggested that there is a complex interaction between VEGF and TGF-β1, both of which promote angiogenesis but have opposite effects on ECs. VEGF protects ECs against apoptosis, whereas TGF-β1 induces apoptosis. Moreover, the suppression of VEGF inhibited both the angiogenesis and apoptosis induced by TGF-β1, suggesting a refined regulation of angiogenesis through the interaction of these growth factors (Ferrari et al., 2009). In the injured brain, the application of TGF-β distinctly facilitated the re-elongation of axons in the injured site in a concentration-dependent manner, suggesting that TGF-β1 can also facilitate axonal regeneration of injured brain neuronal cells (Abe et al., 1996).

### GM-CSF/G-CSF

GM-CSF is known to stimulate the growth and polarization of granulocytes and macrophages, exerting a crucial effect in the regulation of innate and adaptive immunity (Seledtsov et al., 2019). As a potent survival factor for monocytes/macrophages, GM-CSF may promote arterial formation in patients with cerebral ischemia to enhance their collateral capacity, thereby reducing the brain infarction risk (Lee et al., 2005; Maurer et al., 2008). A previous clinical trial has shown that GM-CSF has a positive effect on the therapeutic enhancement of angiogenesis in a group of patients with coronary artery disease (Werner, 2002). Previous research results of GM-CSF subcutaneous injection in hypoperfused rat brain showed that: (1) GM-CSF induced an enlargement of posterior cerebral artery caliber on three-vessel occlusion (3-VO, a non-lethal brain hypoperfusion model); (2) morphological and functional changes could be caused by subcutaneous GM-CSF treatment; (3) GM-CSF improved the cerebral hemodynamic parameters on 3-VO, such as CO_2_ reactivity; and (4) GM-CSF enhanced the infiltration of monocytes/macrophages in the area of vascular collateral formation (Buschmann et al., 2003). These facts indicate that GM-CSF treatment can induce arteriogenesis in hypoperfused rat brain.

G-CSF activates the survival, multiplication, and maturation of cells in the neutrophil lineage (Malashchenko et al., 2018). It is also a neuroprotective cytokine and has a positive effect on angiogenesis and neurogenesis after IS. G-CSF plays a role in regulating the PI3K/AKT signaling pathway, affecting cell proliferation and apoptosis and inducing VEGF expression (Liang et al., 2018). G-CSF increases EC multiplication, the vasal branch points, the vascular surface area, and the length of blood vessels in the ischemic penumbra region (Lee et al., 2005). In addition, the neuroprotective action of G-CSF may be confirmed from all the decreases of the infarct size, the inflammatory infiltration, the BBB disruption, and the hemispheric atrophy after G-CSF treatment (Menzie-Suderam et al., 2020; Modi et al., 2020). Two possible molecular mechanisms deserve to be considered in angiogenesis induced by G-CSF: (1) the direct activation of cerebral ECs (Liang et al., 2018) and (2) the transference of bone marrow endothelial progenitor cells (EPCs) to the ischemic penumbra area. G-CSF was confirmed to stimulate ECs in order to initiate an activation/differentiation procedure, including multiplication and migration, associated with angiogenesis (Bussolino et al., 1991). Moreover, evidences demonstrate that G-CSF increases the expression of angiopoietin-2 (Ang2) and endothelial nitric oxide synthase (eNOS) in the ischemic penumbra of the cerebral cortex (Lee et al., 2005). eNOS is known to be essential to increasing both angiogenesis and the number of circulating EPCs (Endres et al., 2004), while Ang2 has been shown to promote a rapid proliferation of capillary diameter, the basal lamina reestablishment, and the development of new vessels (Ramsauer and D'Amore, 2002).

### FGF

Presently, the FGF family is known to contain more than 20 factors. FGFs are secreted by ECs and are presented in the ectocytic matrix. FGFs are affected by different factors in different tissues. The members of the FGF family exert critical roles in angiogenesis for new blood vessel formation, which dramatically affects the proliferation, mobilization, metastasis, and mobilization of vascular ECs (Chen M. et al., 2020). In particular, FGF-1 (acidic FGF) and FGF-2 (basic FGF) have been verified to affect the modeling of new vessels in some tissues *in vivo* by regulating ECs (Zhou et al., 2009; Oladipupo et al., 2014). Moreover, FGF-1 and FGF-2 may restrain endothelial-dependent monocyte recruitment and thus have an anti-inflammatory function during angiogenesis in chronic inflammation (Zhang and Issekutz, 2002). Previous researches have illustrated that FGF-1 can initiate angiogenesis and neurogenesis in rats following IS (Cheng et al., 2011) and protect hippocampal neuronal cells against apoptosis induced by IS (Cuevas et al., 1998). Additionally, in mice after IS and human brain microvascular ECs after oxygen–glucose deprivation (OGD), non-mitogenic FGF-1 enhanced angiogenesis through regulating sphingosine-1-phosphate 1 (S1P1) signaling mediated by the activation of FGF receptor 1 (FGFR1) (Zou et al., 2020). FGF-2 also plays crucial roles in neurogenesis, angiogenesis, and neuronal survival as well as BBB protection. The expression of FGF-2 in the ischemic penumbra zone of all patients was distinctly increased compared with the infarcted tissue and normal contralateral hemisphere (Issa et al., 2005). Upregulated FGF-2 expression was observed to enhance endogenous neovascularization and hemodynamic changes in the infarct region and to upregulate the number of neuroblast cells migrating toward the ischemic striatum (Oyamada et al., 2008). Moreover, the function of promoting angiogenesis and BBB protection effect as well as the neuroprotective function of FGF-2 are partially via relieving the OGD/R-induced reduction of S1P1 receptor 1 (S1PR1) (Lin et al., 2018), binding of FGFR1 and activating the ERK signaling pathway (Chen P. et al., 2020), and regulating via the PI3K–AKT–Rac1 pathway (Wang et al., 2016).

### HGF

HGF is a multifunctional growth factor that plays critical roles in mitogenesis, morphogenesis, motogenesis, anti-apoptosis, and angiogenesis. In the brain, HGF functions as an angiogenic factor and a neurotrophic factor. In addition, HGF exhibits neuroprotective actions on ischemia-induced damage through promoting angiogenesis, neurogenesis, and synaptogenesis and inhibiting fibrotic change (Shang et al., 2011). The application of dental pulp stem cells overexpressing HGF in the acute stage of stroke enhanced their neuroprotective actions through regulating inflammation and BBB permeability, thereby mitigating cerebral ischemia–reperfusion injury (CIRI) (Sowa et al., 2018). HGF may also be involved in the prognosis of acute IS at 3 months (Zhu et al., 2018).

### PDGF

The PDGF family is composed of four elements: PDGF-A, PDGF-B, PDGF-C, and PDGF-D. PDGF is known to be angiogenic and neuroprotective in IS where PDGFR-α activation in circumvascular astrocyte cells is involved in the acute opening of the BBB. Evidence shows that PDGF-B and its receptor on microvessel ECs may be involved in angiogenesis following IS (Krupinski et al., 1997). Deficiency of either PDGF-B or PDGFR-β is closely related to characteristics of vascular rupture, including vascular dysfunction, pericyte defects, and the formation of microaneurysms (Gaengel et al., 2009). PDGFR-β expression is low in adult normal matured brain. However, its expression gradually increases mainly in pericytes and fibroblast-like cells in the ischemic penumbra zone after IS. PDGFR-β mediates a variety of functions, including the modulation of the BBB and the rescue of infarcted tissues after IS (Nakamura et al., 2016). Moreover, a current study has shown critical actions of PDGF-C and PDGF-D in neovascularization, fibrosis, atherosclerosis, and restenosis (Folestad et al., 2018).

### Interleukins

#### IL-1

IL-1 is known to be a primary mediator of central and peripheral inflammation after IS, primarily via the action of both of its subtypes, IL-1α and IL-1β, at cerebral ECs. Previous preclinical studies have demonstrated the deleterious actions of IL-1 following IS, while blocking its actions is salutary in preclinical and clinical settings. However, the pro-inflammatory cytokine IL-1α was found to increase the brain EC activation, migration, and the proliferation of ECs and to induce chemokine (C–X–C motif) ligand 1 (CXCL-1) and IL-6 (recognized to have angiogenic effects in cerebral ECs) expression as well as promote the construction of capillary tube-like structures in cerebral ECs *in vitro* (Salmeron et al., 2016). Hence, these findings demonstrate that IL-1α has angiogenic effects in post-stroke angiogenesis. After ischemia injury, oligodendrocytes respond to inflammatory IL-1β signaling and produce MMP-9 to enhance angiogenesis (Pham et al., 2012).

#### IL-4

As an important anti-inflammatory cytokine, IL-4 induces mononuclear/macrophages to differentiate into M2 macrophages through its receptor IL-4R and then secretes more anti-inflammatory factors such as IL-10 and TGF-β, while inhibiting the formation of M1 macrophages and the release of TNF-α and IL-1 (Bhattacharjee et al., 2013). M2 macrophages are known to promote angiogenesis after IS, partially through regulating the HIF1-α/VEGF/NO signaling pathway. Evidence shows that the blockade of IL-4 abrogates the promoting role of hyperforin in post-stroke angiogenesis, neuronal regeneration, and functional rehabilitation (Yao et al., 2019).

#### IL-6

Four weeks after MCAO, IL-6 knockout mice displayed a distinctly elevated chronic lesion size and showed a damaged angiogenic response to cerebral ischemia with the reduction of neonatal EC numbers, perfused microvessel density, absolute focal cerebral blood flow, and vessel responsivity in ischemic striatum. These findings suggest that IL-6 plays an important role in angiogenesis after IS. Moreover, resident cerebral cells are the main sources of elevated levels of IL-6 in the early-phase post-stroke. IL-6 stimulates the phosphorylation of signal transducer and activator of transcription 3 (STAT3) and the early transcriptional activation of angiogenesis relative genes, thereby leading to the enhanced angiogenesis and increased brain blood flow in the delayed phase after IS (Gertz et al., 2012). IL-6R simultaneously activates the PI3K/AKT and Janus kinase (JAK)/STAT signaling pathways (Xu et al., 2005), which play vital roles in angiogenesis after stroke.

## The Role of Inflammation-Relative Signaling Pathways in Post-stroke Angiogenesis

### JAK2/STAT3 Pathway

**Figure 1:**
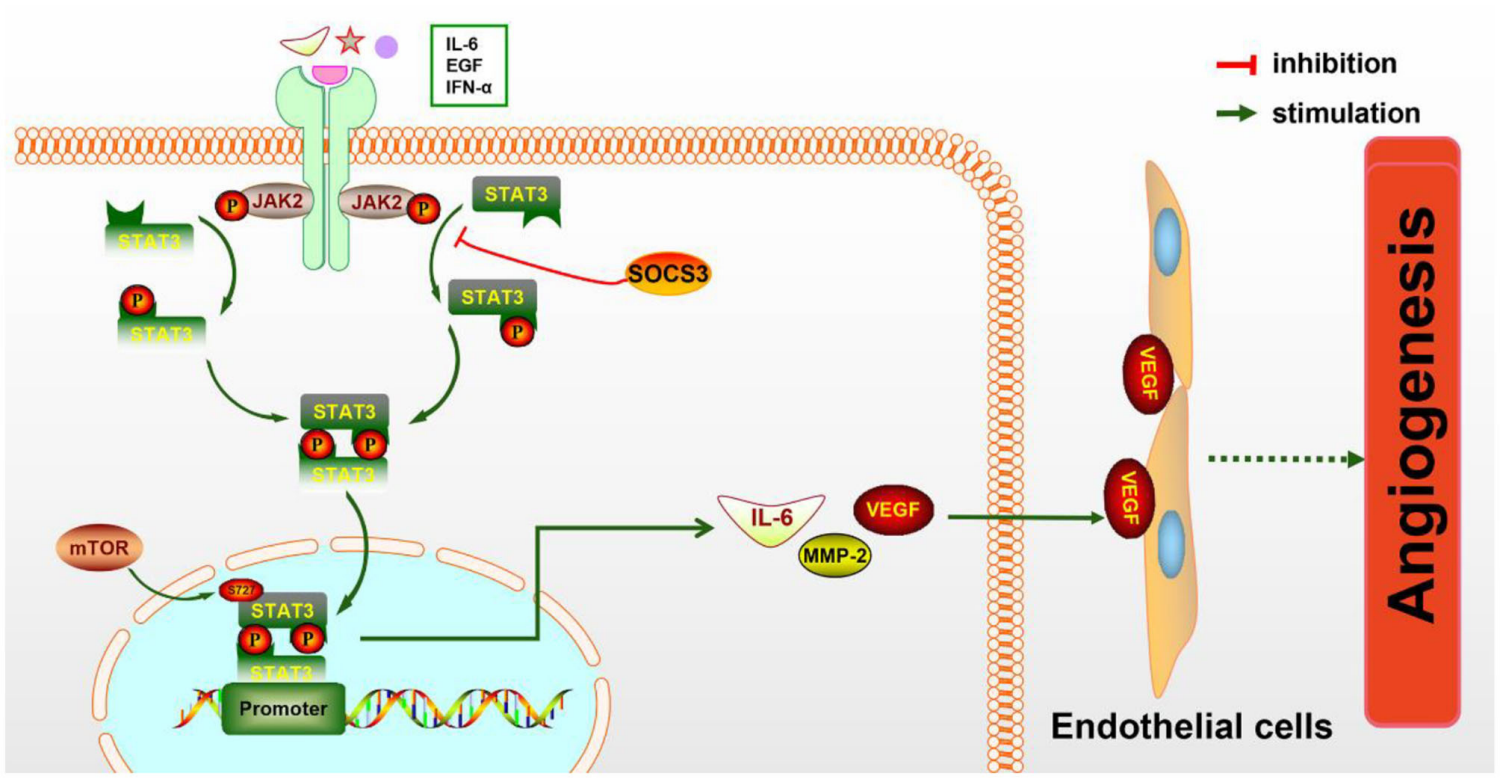
The molecular mechanism of angiogenesis mediated by the JAK2/STAT3 pathway. JAK2 is activated and phosphorylated by inflammatory cytokines such as IL-6 and IFN-α, resulting in the phosphorylation of downstream STAT3 and the formation of the p-STAT3 dimer, which is subsequently transferred to the nucleus to promote the expression of particular genes. IL-6, MMP-2, VEGF, and other cytokines are increased, then secreted to the extracellular matrix and act on endothelial cells (ECs), eventually leading to increased angiogenesis.

STAT3 is known to be a key regulator of various inflammatory signals. STAT3 is phosphorylated by phospho-JAK2 (p-JAK2) and is subsequently transferred into the nucleus to regulate the expression of particular genes. Numerous evidences have shown that JAK2/STAT3 signaling activation plays a protective effect against CIRI (Hoffmann et al., 2015; Li et al., 2017). As a direct transcriptional activator of VEGF under hypoxia conditions, STAT3 has recently been confirmed to regulate post-stroke angiogenesis, axon growth, and extracellular matrix remodeling (Hoffmann et al., 2015). Moreover, deficiency of STAT3 signaling is involved in the inhibition of pancreatic carcinoma and kidney cancer through suppressing cancerous angiogenesis (Li et al., 2013). The potential molecular mechanism of angiogenesis induced by the JAK2/STAT3 signaling pathway may be the induction of VEGF production and the regulation of Ang-1 and Ang-2 expression (Li et al., 2017) (Figure 1). As is known, Ang-1 promotes the maturity of new blood vessels and Ang-2 is a destabilizing signaling that reverts vessels to a more malleable state. Numerous cytokines and agents can promote STAT3 phosphorylation or directly activate the JAK2/STAT3 signaling pathway, including IL-6, secretoneurin (Shyu et al., 2008), salvianolic acids (Li et al., 2017), catalpol (Dong et al., 2016), ginkgolide K (Chen et al., 2018), etc., exerting an angiogenic action. What is more, the activation of the JAK2/STAT3 signaling pathway can shift the microglia toward M2 polarization (Qin et al., 2017), which promotes angiogenesis in IS. Additionally, the STAT3 signaling pathway is essential for HIF-1α expression regulated by VEGF and PI3K/AKT. The expressions of HIF-1α and VEGF disappeared after STAT3 was blocked by its inhibitor, such as CPA-7 or STAT3 small interfering RNA (siRNA) (Xu et al., 2005). However, the inhibition of STAT3-dependent autophagy can also promote angiogenesis after IS (Xia et al., 2020).

### PI3K/AKT Signaling Pathway

The PI3K/AKT pathway is known to exert a crucial effect in various physiological conditions such as inflammation, oxidative stress, and apoptosis. The activation of the PI3K/AKT pathway can decrease the expression of inflammatory genes and then maintain the vascular capacity. Furthermore, the PI3K/AKT signaling pathway promotes neuronal survival activation and enhances cell survival after IS (Zhang et al., 2016; Li et al., 2019). Activated AKT can promptly stimulate some molecular signals such as rapamycin (mTOR), which promotes protein synthesis and regulates cytotrophy and energy supply. A previous study has illustrated that the activated PI3K/AKT/mTOR pathway exerts essential actions on the effect of apoptosis and inflammation in the brain (Li et al., 2015).

**Figure 2:**
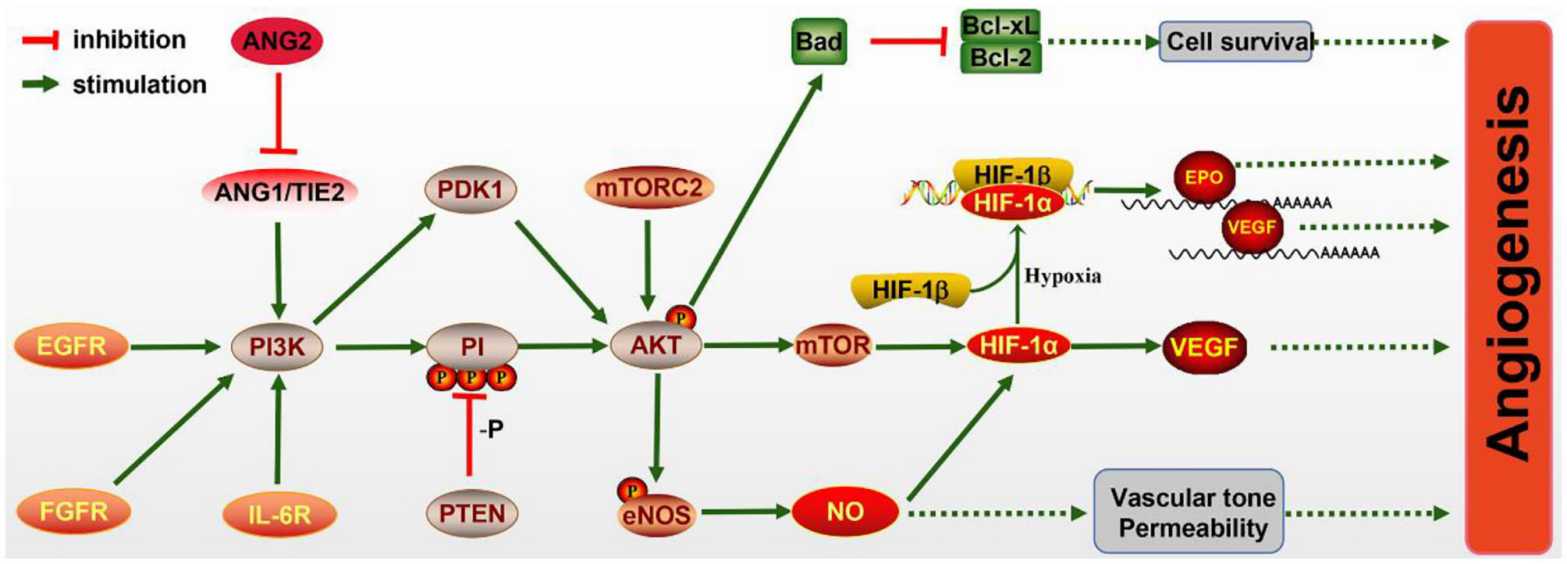
The molecular mechanism of angiogenesis mediated by the PI3K/AKT pathway. PI3K signaling is activated by the endothelial growth factor receptor (EGFR), fibroblast growth factor receptor (FGFR), IL-6R, and Ang/Tie2 signaling molecules, leading to AKT activation. Activated AKT promotes the expression of Bad, which inhibits Bcl-CL/Bcl-2, thereby promoting the survival of pro-angiogenic cells. The mechanistic target of rapamycin (mTOR) is also activated by AKT and increases the level of HIF-1α, which can directly increase the expression of vascular endothelial growth factor (VEGF) or bind to HIF-1β in hypoxia conditions and transfer to the nucleus to promote the expressions of erythropoietin (EPO) and VEGF. Meanwhile, endothelial nitric oxide synthase (eNOS) activated by AKT promotes the production of NO, which protects vascular tone permeability. These potential mechanisms contribute to angiogenesis through the PI3K/AKT pathway.

What is noteworthy is that the PI3K/AKT/mTOR pathway has been proven to be essential for angiogenesis (Figure 2), including EC survival, migration, and new vessel formation. The activated PI3K/AKT/mTOR signaling activates its downstream factor HIF-1α, thereby regulating VEGF expression (Patra et al., 2019), which is known to enhance angiogenesis after IS. In addition, epidermal growth factor (EGF) exerts neuroprotective effects on cerebral ischemia via activating the EGF receptor (EGFR), which also contributes to the activation of the PI3K/AKT/mTOR pathway and elevated HIF-1α (Karar and Maity, 2011).

In ECs, the PI3K/AKT pathway participates in angiogenesis via regulating nitric oxide (NO) signaling, which is modulated by the enzyme NO synthase (NOS). Evidence has illuminated that NO donors can promote the transcriptional activity and expression of HIF-1, thereby inducing the VEGF mRNA (Papapetropoulos et al., 1997). Furthermore, VEGF can initiate NO generation, which is suppressed by the PI3K inhibitor. Hypoxia can also accelerate the phosphorylation of eNOS via activating the PI3K/AKT pathway and HSP9O binding to eNOS. NO production may also be induced *via* the phosphorylation of eNOS by AKT (Kasuno et al., 2004).

The Ang1/Tie2 system, including the angiopoietins and their receptors, can also activate the PI3K/AKT pathway and promote EC survival. Ang1 and Ang2 bind to Tie2 receptor tyrosine kinase, which is mainly expressed in ECs (Davis et al., 1996; Karar and Maity, 2011). Ang1 is essential for endothelial development, while Ang2 resists the Ang1/Tie2 system and can disrupt vascularization. Research has shown that Ang2 can activate the PI3K/AKT pathway and serve as a stimulant of the Tie2 receptor in the deficiency of Ang1, although weaker than Ang1 (Yuan et al., 2009).

### MAPK Pathways

MAPK pathways amplify, transmit, and integrate signals from a various range of stimuli and induce appropriate physiological reactions including proliferation, differentiation, development, transformation, apoptosis, and inflammatory responses. To date, three MAPK families have been characterized: ERK, p38 MAPK, and c-Jun N-terminal kinase/stress-activated protein kinase (JNK/SAPK). The activation of p38 MAPK/HIF-1α/VEGF-A exerts anti-apoptotic and angiogenic effects against CIRI (Cheng et al., 2017). The application of MAPK/ERK pathway inhibitors disrupts VEGF-induced angiogenesis, thereby indicating that the MAPK/ERK signaling pathway mediates this process (Pulous et al., 2020). Moreover, hyperbaric oxygen-induced VEGF is activated by C-Jun/ AP-1 and through simultaneously activating the JNK and ERK signaling pathways (Lee et al., 2006). These findings indicate that ERK, p38 MAPK, and JNK are all involved in regulating angiogenesis in different ways.

### NF-κB Signaling Pathway

NF-κB, a typical pro-inflammatory signaling molecule, is a key transcriptional factor that plays an important role in the regulation of inflammation, angiogenesis, functional circuit formation, and neurite outgrowth (Cui et al., 2015). The expression of angiogenesis-stimulating factors is partially mediated through the transcriptional activation of NF-κB in response to the inflammatory cytokines IL-1 α/β and TNF-α in vascular ECs and monocytes/macrophages, as well as in cancer cells. Inflammatory angiogenesis and the enhanced production of VEGF, in response to IL-1 α/β and TNF-α, are partially due to the activation of the NF-κB pathway (Watari et al., 2012). Furthermore, it has been verified that VEGF application activates NF-κB in vascular ECs. The activation of the NF-κB signaling pathway is through the activation of the PLCγ-sphingosine kinase-PKC cascade, resulting in the upregulation of vascular cell adhesion molecule-1 (VCAM-1) and intercellular adhesion molecule-1 (ICAM-1) in monocytes/macrophages or vascular ECs stimulated by VEGF (Kim et al., 2001). In addition, NF-κB is also essential for the stem cell factor (SCF)+G-CSF-induced angiogenesis in the ipsilateral somato-sensorimotor cortex after stroke (Cui et al., 2015).

## Conclusions and Perspectives

Numerous studies have repeatedly shown that inflammatory reactions play multiphasic and complex roles in the progression and pathogenesis of IS. As mentioned above, pro-inflammatory cytokines such as IL-1β and TNF-α can promote angiogenesis after IS, but too many pro-inflammatory cytokines have a detrimental effect on the progression of IS. Analogously, anti-inflammatory cytokines such as IL-10 exert a protective effect after stroke, but too many anti-inflammatory factors produce immunosuppression after stroke. Thus, a balance between pro-inflammatory and anti-inflammatory signals is urgently needed to improve IS outcomes.

**Figure 3:**
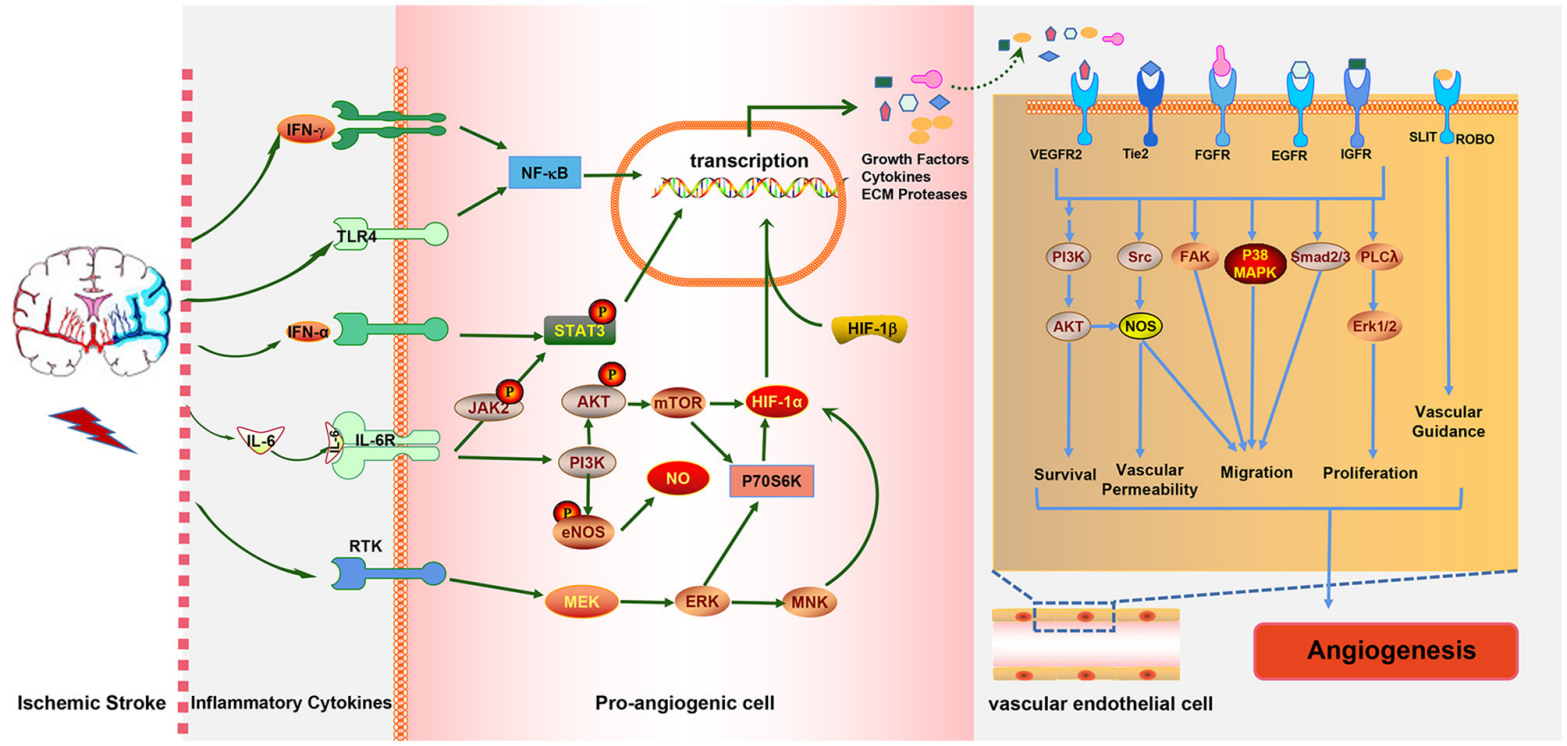
The interaction between ischemic stroke, inflammatory cytokines, and inflammation-associated pathways. Ischemic stroke increases the expressions of the pro-inflammatory cytokines IFN-γ, IL-6 and IFN-α, which activate the NF-κB, JAK2/STAT3, PI3K/AKT, and MAPK pathways in pro-angiogenic cells, leading to the elevated expressions of growth factors, cytokines, and extracellular matrix (ECM) proteases. These cytokines act on endothelial cells (ECs) to promote EC survival, vascular permeability, and migration proliferation and, ultimately, to promote angiogenesis, which in turn alleviates stroke injury.

Various inflammatory cells and inflammatory cytokines, as well as the immune cell subtypes mentioned above, play crucial roles in ischemia-induced angiogenesis. The occurrence of IS induces immune inflammatory responses in brain tissue which can promote angiogenesis. Ultimately, angiogenesis improves stroke prognosis, and eventually, the immune response is mitigated. It is obvious that the three are directly related to each other in a complex way. Moreover, there is also an interaction network between immune cells, inflammatory cytokines, and inflammation-relative signaling pathways involved in post-stroke angiogenesis. For example, IL-6R simultaneously activates the PI3K/AKT and JAK2/STAT3 signaling pathways; in turn, the activation of the JAK2/STAT3 pathway induces the generation of IL-6 (Figure 3). The STAT3 signaling pathway is also essential for the expression of HIF-1α mediated by VEGF and PI3K/AKT, whereas HIF-1α induced by the PI3K/AKT pathway promotes VEGF expression.

In conclusion, in this paper, we mainly discussed the inflammatory and immune cell subsets as well as the associated signaling pathways that contribute to angiogenesis in IS. The immune cell subsets and inflammatory cells involved in angiogenesis after IS mainly contain microglia/macrophage cells, NK cells, CD4^+^ T cells, brain pericytes, ECs, etc. Meanwhile, inflammatory cytokines, such as VEGF, MCP-1/CCL2, TNF-α, TGF-β, GM-CSF/G-CSF, FGF, HGF, PDGF, IL, and HIF-1α, play a critical role in the inflammatory-mediated angiogenesis after IS. Targeting these inflammatory cytokines and immune cells may provide the theoretical basis for potential therapies for IS.

## Author Contributions

All the listed authors directly, substantially, and intellectually contributed to the preparation of this manuscript and approved the publication.

## Conflict of Interest

The authors declare that the research was conducted in the absence of any commercial or financial relationships that could be construed as a potential conflict of interest.
